# Predicting factors of failed induction of labor in three hospitals of Southwest Ethiopia: a cross-sectional study

**DOI:** 10.1186/s12884-021-03862-x

**Published:** 2021-05-19

**Authors:** Amare Genetu Ejigu, Shewangizaw H/mariam Lambyo

**Affiliations:** Department of Midwifery, College of Medicine and Health Science, Mizan-Tepi University, Mizan-Teferi, Ethiopia

**Keywords:** Labor induction, Failed induction, Cesarean section, And Ethiopia

## Abstract

**Introduction:**

Failed induction of labor affects maternal and neonatal outcomes as well as the cost of healthcare, especially in low-resource setting regions in which the prevalence of failed induction is higher despite the incidence of labor induction is low. This study aimed to assess the prevalence of failed induction of labor in southwest Ethiopia.

**Method:**

A hospital-based cross-sectional study was conducted among 441 induced women from March 1 to August 30, 2018. A systematic random sampling technique was used to select study participants. Data were collected using a pretested and structured questionnaire. Bivariable and multivariable logistic regression models were done and fitted to identify predictors of failed induction. An adjusted odds ratio with 95% confidence interval (CI) was calculated to determine the level of significance.

**Result:**

Premature rupture of membrane was the most common cause of labor induction and the commonly used method of labor induction were oxytocin infusion. Cesarean section was done for 28.1% of induced women. Failed induction of labor was found to be 21%. Primiparous [AOR = 2.35 (1.35–4.09)], analgesia/anesthesia [AOR = 4.37 (1.31–14.59)], poor Bishop Score [AOR = 2.37 (1.16–4.84)], Birth weight ≥ 4 k grams [AOR = 2.12 (1.05–4.28)] and body mass index [AOR = 5.71 (3.26–10.01)] were found to be significantly associated with failed induction of labor.

**Conclusion:**

The prevalence of failed induction of labour was found to be high. Preparation of the cervix before induction in primi-parity women is suggested to improve the success of induction. To achieve the normal weight of women and newborns, proper nutritional interventions should be given for women of reproductive age. It is better to use analgesia/anesthesia for labor induction when it becomes mandatory and there are no other optional methods of no- pharmacologic pain management.

**Supplementary Information:**

The online version contains supplementary material available at 10.1186/s12884-021-03862-x.

## Introduction

Induction of labor (IOL) is an interventional designed to artificially initiate uterine contractions [1]. IOL should be done only when there is a clear medical indication for it and the expected benefits outweigh its potential harm [2]. In the past several years, the incidence of labor induction around the world has continued to be raised [2, 3].

Induction of labor is increased risk of poor maternal and perinatal outcomes (perianal laceration, hysterectomy, intensive care unit admission, longer hospital stay, postpartum hemorrhage, and chorioamnionitis) [4–8]. Cesarean delivery is increased approximately 2–3 fold in women who undergo induction of labor compared with spontaneous labor, and failed induction is the most common indication for cesarean delivery [9–18]. Health care expenses for delivery care services were significantly higher for cesarean section (270 USD) than for vaginal delivery (59 USD) [19]. In Ethiopia, the cesarean section is higher among induced women (38.44%) [20] compared to spontaneous labor (19.2%) [21].

Studies were done in American, African, and Asian countries showed that low Apgar scores at 5 min, low birth weight, neonatal intensive care unit admission, stillbirth, and delayed breastfeeding increased when labor induced [4, 8, 22, 23].

The global prevalence of obesity among women has increased from 6% in 1975 to 15% in 2016 [24]. Obese women are at increased risk of impairment of the active phase of labor (specifically dilatation arrest) and prolonged duration of active phase of labor [25, 26]. Maternal obesity and overweight are associated with adverse pregnancy outcomes [27, 28]. Recent studies have shown that maternal obesity increases the risk of obesity in children, affects fetal metabolism and tissue development through the heritage of maternal obesity-susceptible genes and cognitive performance and behavior of the offspring [29–32]. A meta-analysis study revealed that overweight and obese women contributed 21.7 to 41.7% of children overweight/obesity [33].

According to research; maternal age, gestational age, parity, bishop score, PROM, postterm, previous obstetric complications, and birth weight are the most common contributing factors for failed induction [16, 20]. However, the determinants of failed labor induction are not consistent across different health institutions and socio-demographic status across society. Therefore, this study aimed to assess the prevalence of failed induction of labor among induced women in public hospitals in Keffa, Sheka, and Bench Maji Zone, Southwest Ethiopia.

## Methods

### Study setting

The study was conducted at Mizan Tepi University Teaching Hospital, Gebretsadik-shaw General Hospital and Tepi General Hospital from March 1 to August 30, 2018. Those 3 hospitals are the only hospitals which provide induction of labor service in the catchment area. Based on the 2017 Population forecast, a total of 2,218,689 population, of whom 1,123,834 females lived in Bench Maji Zone, Kaffa Zone, and Sheka zones [34]. In the study area, there were 103 governmental health facilities, of which 6 hospitals and 97 health centers. During the study period, only 3 hospitals were provided induction of labor. A Hospital-based cross-sectional study was done among induced women who had a singleton pregnancy, cephalic presentation, longitudinal lie and gestational age 28 weeks and beyond during delivery services.

### Sample size calculation

A single population proportion formula was used to calculate the required sample size of the study. Considering the assumptions of 95% level of confidence, 4% margin of error, and 21.4%(p) the proportion of failed induction of labor, which was taken from a previous study [35]. Considering a 10% nonresponse rate, the final sample size became 449.

### Sampling procedure

A systematic random sampling technique was used to select study respondents. In these three zones, all hospitals that provided induction of labor were included in this study. Distribution of the required sample size was done based on the proportional number of labor inductions performed in each public hospital. The data (the number of labor induction in each public health hospitals) were obtained from delivery register of women prior to actual data collection. Sampling interval approaches were implemented. We calculated the sampling interval (K) using the summation of 6 months of labor induction at public hospitals, which was 1004. Then K = N/n, 1004/449 = 2.23 ≈ 2. Every 2nd induced woman was interviewed and their medical records were reviewed. To start with the first interview, we used lottery method.

### Data collection tool techniques

Data were collected by interviewing women as well as reviewing their medical records. Upon admission and through the period of labor and delivery, information regarding obstetric characteristics, indications for IOL, methods used for IOL, and information about the outcome of IOL were collected. To assure the quality of data, before the actual data collection, technical training was given for data collectors and a pretest was done on 5% of the total sample size outside of the study area, which has similar characteristics to the study population. To collect all the required sample sizes, we used 6 trained diploma midwives as data collectors and 2 physicians for supervisors. The supervisors checked each filled questionnaire for completeness, accuracy, and consistency daily.

### Data analysis

After data were collected, the questionnaires were coded and entered using Epi data version 3.1 and exported to SPSS software version 20 for analysis. Descriptive statistics were carried out to characterize the study population using different variables. Variables with a *p*-value < 0.2 in the bivariate analysis were entered into the multiple logistic regressions for further analysis. Finally, variables with a *P* value < 0.05 in the multivariable logistic analysis were considered statistically significant.

### Operational definition

#### Failed induction

is defined as failure to achieve regular (e.g. every 3 min) uterine contractions and cervical change after at least 6–8 h of the maintenance dose of oxytocin administration, with artificial rupture of membranes. Artificial rupture of membranes is done for induction of labor with alive fetus. Artificial rupture of membranes is not done for induction of labor indicated with Intra-Uterine Fetal Death.

#### Post-term

is defined as a pregnancy that advances to or beyond 42 completed weeks or 294 days of gestation from the first day of the last normal menstrual period.

### Protocol and implementation of induction of labor

In the study area (in all hospitals) both mechanical (balloon catheter and Sweeping membrane) and medical (misoprostol and oxytocin) methods are employed for induction of labor depending on the favorability of the cervix. When the cervix becomes unfavorable (bishop’s score < 4), 25 μg vaginal misoprostol is given in 6 h intervals and if there is no response, the dose of misoprostol is escalated to a maximum of 200 μg for cervical ripening. Sometimes women go to the active phase of labor with misoprostol before oxytocin infusion [36]. Induction of labor in our study setting follows the national guideline protocol in which 5 IU of oxytocin is added into 1000 ml of N/S or R/L solution and adjust the number of drops every 30 min. Induction of labor starting with a low dose of oxytocin and increase every 30 min till adequate uterine contraction is achieved (Table 1, supplementary material).

**Table 1:** Protocol and schedule used for escalating oxytocin dosage induction of labor in the study area

Dose and oxytocin concentration	Time	Drops / minute 1 ml ≈ 20 drops	Approximate oxytocin in mIU/ minute
First dose: 5 IU of oxytocin in 1000 ml fluid	0:00 h	20	2
	0:30 h	40	4
	1:00 h	60	6
	1:30 h	80	8
Second dose: Add another 5 IU of oxytocin to the remaining first dose fluid	2:00 h	50	12
	2:30 h	60	15
	3:00 h	80	20
Third dose: Add another 5 IU of oxytocin on the remaining second dose fluid	3:30 h	50	24
	4:00 h	60	30
	4:30 h	80	40
	5:00 h	As above	As above
	5:30 h	As above	As above

*mIU* Million international unit, *N/S* Normal saline and *R/L* Ringer lactate

## Results

### Socio-demographic characteristics of participants

A total of 441 induced women were interviewed with a response rate of 98.2%. One hundred ninety-one (43.3%) women were in the age group of 24–28 with a mean age was (25.8 years + 4.89SD), 231(52.4%) women were orthodox Christian, and 247(56%) mothers lived in urban areas (Table 2).

**Table 2:** Socio-demographic and obstetric characteristics of induced women in public hospitals of Keffa, Sheka and Bench-Maji Zone, Southwest Ethiopia, 2018

Variables	Categories	Frequency (*n* = 441)	Percent (%)
Age	19–23	143	32.4
	24–28	191	43.3
	29–33	69	15.6
	≥34	38	8.7
Residence	Urban	247	56
	Rural	194	44
Religion	Orthodox	231	52.4
	Protestant	112	25.4
	Muslim	78	17.7
	Catholic	20	4.5
Marital status	Married	414	93.9
	Not married	21	4.8
	Widowed	4	0.9
	Divorced	2	0.5
Educational status	Unable to read and write	84	19
	Able to read and write	133	30.2
	Primary (1–3, 5–9)	96	21.8
	Secondary and above	128	29
Occupational status	Gov’t Employee	65	14.7
	House wife	232	52.6
	Merchant	70	15.9
	Others specify^a^	74	16.8
Ethnicity	Bench	92	20.9
	Kafficho	186	42.2
	Shakacho	94	21.3
	Amhara	34	7.7
	Oromo	19	4.3
	Tigre	6	1.4
	Others specify^b^	10	2.3
Parity	Primi	227	51.5
	Multi	214	48.5
Gestational age	28–36	82	18.6
	37–41	258	58.5
	≥42	101	22.9
Last child alive (*n* = 214)	Yes	196	91.6
	No	18	8.4
Previous obstetric complications (*n* = 214)	Yes	24	11.3
	No	190	88.7
Analgesia/anesthesia	Yes	16	3.6
	No	425	96.4
Pre-induction bishop score	≤5	310	70.3
	> 5	131	29.7
Body mass index (kg/m^2^)	≤24	337	76.4
	> 24	104	23.6
Cervical dilatation (cm)	1–2	326	73.9
	> 2	115	26.1

^a^ Student, daily labor ^b^ Guarage

### Obstetric characteristics of participants

Two hundred fourteen (48.5%) women in the study were multipara**,** 258 (58.5%) of pregnancies were found in the gestational age category of 37–41 weeks. The mean fetal gestational age was 38.73 (SD ± 2.72). Of the 214 multiparous induced women, 24(11.3%) had previous obstetric complications and 18(8.4%) women were lost their last child. The Bishop scores of 310(70.3%) study participants were ≤ 5 before the induction of labor (Table 2).

### Methods and indications for labor induction

One hundred forty-five (32.9%) women were induced for indication of PROM, 307 (69.6%) mothers were induced with oxytocin infusion and 246 (55.8%) mothers were induced with misoprostol (vaginal and/or oral route) (Table 3).

**Table 3:** Indication and method of labor induction among women delivered in public health hospitals of Keffa, Sheka and Bench-Maji Zone, South West Ethiopia, 2018

Variable	Category	Frequency	Percent (%)
**Indication**	Post term	101	22.9
	Premature rapture of membrane	145	32.9
	Hypertensive disorder	116	26.3
	Diabetes mellitus	5	1.1
	Intra uterine growth restriction	11	2.5
	Ante partum hemorrhage	48	10.9
	Others ^a^	44	10
**Methods of Induction**	Balloon catheter	51	11.6
	Sweeping membrane	1	0.2
	oxytocin infusion	307	69.6
	Misoprostol	246	55.8

^a^ = Oligohydramnios, intrauterine fetal death

### Outcomes of labor induction

**Fig. 1:**
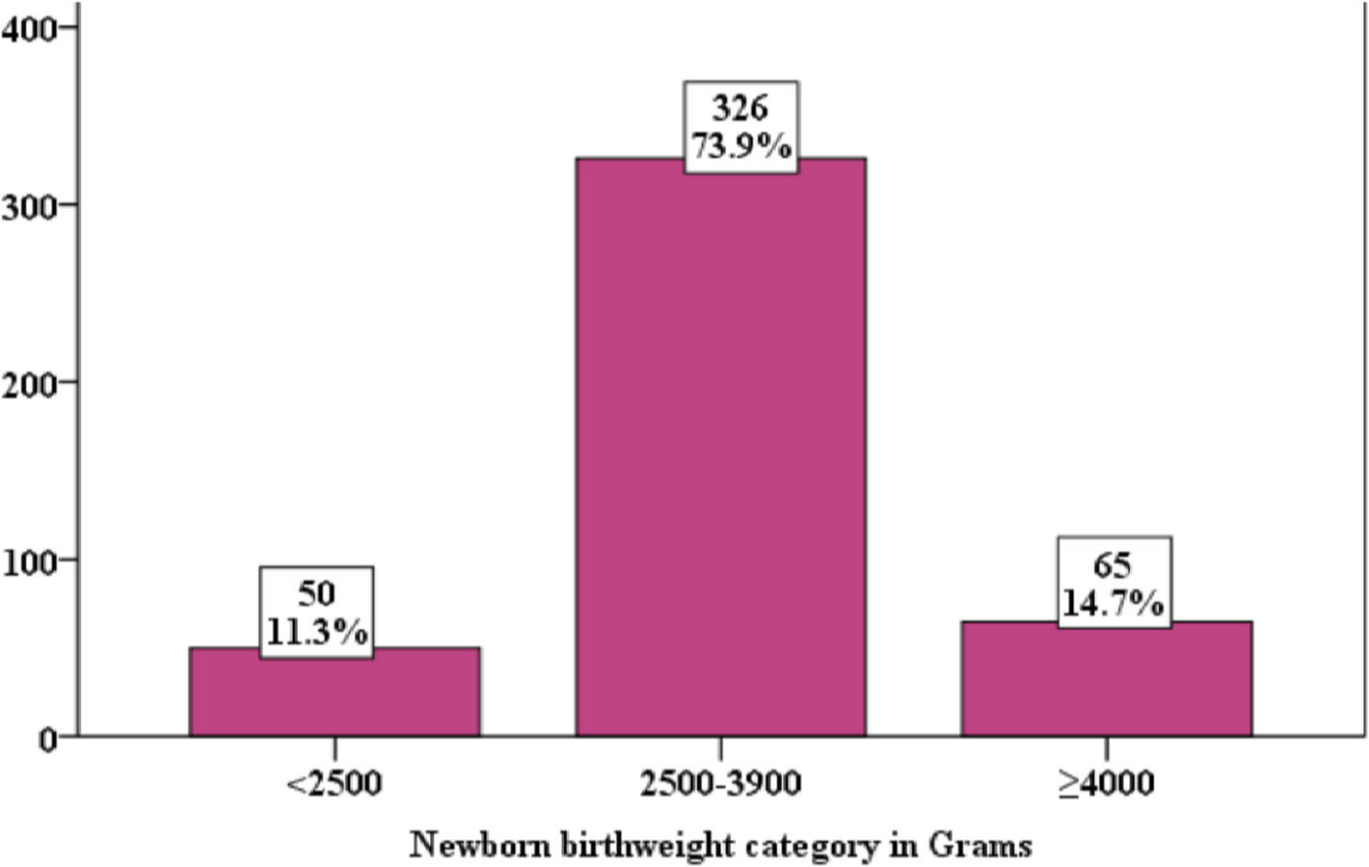
Newborn brith weight of induced women in public health hospitals of Keffa, Sheka and Bench-Maji Zone, South Ethopia, 2018

Two hundred forty-three (55.1%) mothers gave normal vaginal birth, and 124 (28.1%) mothers gave birth through cesarean section. Nearly two-thirds (64.5%) of the cesarean section was done due to failed induction of labor**.** The rest cesarean section cases were done for indication of fetal distress and cephalo-pelvic disproportion. For those cases cesarean section was done before the status of labor induction (failed or succeed) was determined. Fifty-four (10%) induced women experienced uterine hyper-stimulation, 90(20.4%) induced women faced fetal heart rate nonreassuring, 8 (1.8%) induced women encountered uterine rupture, 57 (12.9%) induced women end up with stillbirth, 169 (38.3%) and 97 (22%) newborns had Apgar scores < 7 at 1st and 5th minutes, respectively (Table 4). Three hundred twenty-six (73.9%) mothers had given weight of 2500–3900 g newborn (Fig. 1).

**Table 4:** Outcomes of inductions of labor among women delivered in public health hospitals of Keffa, Sheka and Bench-Maji Zone, South West Ethiopia, 2018

Variables	Category	Frequency (*n* = 441)	Percent (%)
Mode of delivery	Normal vaginal delivery	243	55.1
	Assisted vaginal delivery	74	16.8
	Caesarean section	124	28.1
Reason for Caesarean section (*n* = 124)	Failed induction	80	64.5
	CPD	24	19.3
	Fetal distress	51	41.1
Uterine hyper stimulation	Yes	44	10
	No	397	90
Uterine rupture	Yes	8	1.8
	No	433	98.2
Fetal heart rate non-reassuring	Yes	90	20.4
	No	351	79.6
Stillbirth	Yes	57	12.9
	No	384	87.1
APGAR score less than 7	At 1 min	169	38.3
	At 5 min	97	22

### Failed induction of labor

**Fig. 2:**
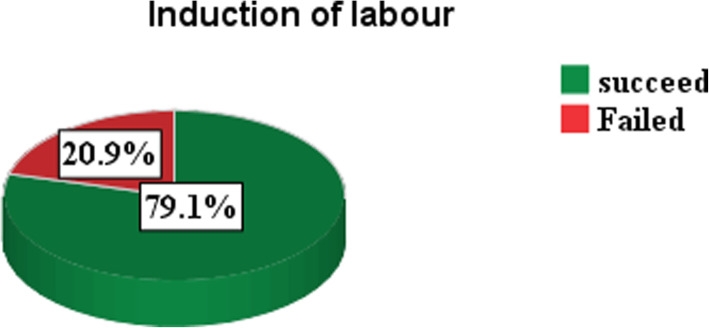
Distribution of induction of labor among women in public health hospitals of Keffa, Sheka and Bench-Maji Zone, South West Ethiopa, 2018

In this study, ninety-two (20.9%) of the study subjects had failed induction of labor (Fig. 2).

### Factors associated with failed labor induction

Pre-induction bishop scores ≤5, birth weight ≥ 4000 g, Primiparous, analgesia/anesthesia and body mass index (kg/m^2^) > 24 were significant factors for failed induction of labor. The odds of induced women whose pre-induction bishop score ≤ 5 were 2.3 times more likely to have failed induction [AOR = 2.37 (1.16–4.84)]. Women whose newborn birth weight ≥ 4000 g were 2 times as likely to have failed induction as compared with women whose newborn birth weight was 2500–3900 g [AOR = 2.12 (1.05–4.28)]. The odds of failed induction were 2.3 times more likely in Primiparous [AOR = 2.35 (1.35–4.09)] than in multiparous women. The odds of failed induction were 4.3 times more likely among women who were given analgesia/anesthesia [AOR = 4.37 (1.31–14.59)] than mothers who were not given. Women whose body mass index > 24 kg/m^2^ were 5.7 times more likely to have failed induction as compared to their counterparts [AOR = 5.71 (3.26–10.01)] (Table 5).

**Table 5:** Factors associated with failed induction of labor in public hospitals of Keffa, Sheka and Benchi-Maji Zone, Southwest Ethiopia, 2018

Variables		Failed induction of labor				
		Yes (***n*** = 92)	No (***n*** = 349)	COR (95.0%CI)	AOR (95.0%CI)	***P***-value
Parity	Multi	30	184	1		
	Primi	62	165	2.30 (1.42, 3.73)	2.35 (1.35,4.09)^a^	.002
Premature rapture of membrane	Yes	23	122	.62 (.36, 1.04)	1.06 (0.54,2.08)	.857
	No	69	227	1		
Body mass index (kg/m^2^)	≤24	43	294	1		
	> 24	49	55	6.09 (3.69,10.04)	5.71 (3.26,10.01)^a^	.000
Cervical dilatation (cm)	1–2	82	244	3.52 (1.76,7.07)	2.30 (0.99,5.29)	.050
	> 2	10	105	1		
Analgesia/anesthesia	Yes	9	7	5.29 (1.91, 14.63)	4.37 (1.31,14.59)^a^	.016
	No	83	342	1		
Bishop score	≤5	77	233	2.55 (1.40,4.64)	2.37 (1.16–4.84)^a^	.017
	> 5	15	116	1		
Newborn birth weight	< 2500	6	44	0.63 (0.25,1.54)	0.57 (0.18,1.76)	.334
	2500–3900	58	268	1		
	≥4000	28	37	3.49 (1.98,6.16)	2.12 (1.05,4.28)^a^	.035
Gestational age	28–36	14	68	.92 (0.47,1.78)	0.98 (0.42, 2.24)	.963
	37–41	47	211	1		
	≥42	31	70	1.98 (1.17,3.37)	1.71 (0.83,3.51)	.141

^a^ = *P* < 0.05: Statistically significantly associated

## Discussion

This study revealed that failed induction of labor was found to be 20.9% (95% CI: 17.5, 24.7). This is in line with studies done in Nigeria (24.1%), WHO survey in African and Asian countries (20%), southwest Ethiopia (21.4%), and Israel (21.6%) [22, 35, 37, 38].

This finding is higher than studies done in America (15.7%) [39] and southern Ethiopia (17.3%) [20]. This difference might be due to variation in methods for induction of labor, oxytocin infusion was a commonly used method in the present study, while in the other studies, misoprostol with a balloon catheter was used as a common practice [37]. This might also be due to differences in indications for induction of labor. In our study, PROM was the most common indication in another study, elective and hypertension [8]. This is lower than that done in Latin America, 29.6% [8]. This might because there is a high rate of labor induction in high-income countries as compared to low-income countries [3, 22].

Primiparous women were 2.3 times more likely to had failed induction than multipara women. This study is similar to the previous studies; nulliparous women are at greater risk for both failed inductions and cesarean sections as compared to multiparous women [12, 16, 20, 35, 38, 40, 41]. This might be due to the higher proportion of unfavorable bishop scores in Primiparous in the study; it may also be the reason that the lack of important practices likes the use of misoprostol with oxytocin to induction in Primiparous.

Women whose body mass index > 24 kg/m^2^ were 5.7 times more likely to had failed induction as compared with women whose body mass index ≤24 kg/m^2^. This finding is supported by previous findings [38, 39, 41–43]. This could be due to; maternal obesity is related with a lower bishop score, women with lower bishop scores are at greater risk for failed induction [41]. In addition, to achieve vaginal delivery, obese women require more concentration, higher doses, and longer duration of exposure of uterotonics medication, using similar protocol and guidelines on labor induction for all women with different BMI to end up with higher failure rate among obese women [44, 45]. The current study has been shown that uterine contractility impairment is higher among morbid obesity women; uterine contractility dysfunction might lead to failed induction [46].

Women who were given analgesia/anesthesia were 4.3 times more likely to had failed induction as compared to women who were not given analgesia/anesthesia. This finding is supported by studies in which the earlier epidural analgesia was given during labor, the higher the probability of cesarean delivery among induced women [7, 13, 18]. This might be using epidural anesthesia during induction of labor was related to a low bishop score which increased the rate of failed induction [41].

Mothers whose pre-induction bishops score ≤ 5 were 2.3 times more likely to have failed induction as compare to mothers whose pre-induction bishop score > 5. This finding is comparable with the studies [20, 35, 38, 40].

Women whose newborn birth weight ≥ 4000 g were 2 times more likely to have failed induction as compared with women whose newborn birth weight was 2500–3900 g. This study is supported by the previous studies [16, 39]. This might happened due to the mean birth weight of neonates were higher among women who had greater BMI [44], larger BMI leads to lower bishop scores, and failed induction of labor [41].

### Limitation of the study

The economic situation of participants might be one of the causes that could affect the failed induction of labor. However, majority of the women who were participated in this study were house wives and did not remember the economic status the family. Some of the study variables had wider CI and this might not happened if the sample size was larger.

## Conclusion

The prevalence of failed induction of labor was high in the study area. Variables that increased the likelihood of failed induction were body mass index > 24 kg/m^2^, bishop score ≤ 5, Primiparous, birth weight ≥ 4000 g, and using analgesia/anesthesia. Preparation of the cervix before starting induction in Primiparous women is recommended to improve the success of induction. To achieve the normal weight of women and newborns, proper nutritional interventions should be given for women of reproductive age. Consider the risk of failed induction in case of the provision of analgesia/anesthesia.

## Supplementary Information

**Additional file 1.**

## Data Availability

When an ethics statement was obtained from the zonal health office, hospitals, and women, we had agreed and signed not to publish the raw data retrieved from the induced women. However, the datasets collected and analyzed for the current study are available from the corresponding author and can be obtained on a reasonable request.

## Abbreviations

AOR: Adjusted Odd Ratio
CI: Confidence Intervals
IOL: Induction of Labor
PROM: Prelabor Rupture of the Membranes
SPSS: Statistical Package for Social Scientists
USD: United States dollar
