# Supplementary material for: Predicting factors of failed induction of labor in three hospitals of Southwest Ethiopia: a cross-sectional study
Source: BMC Pregnancy Childbirth. 2021 May 19;21:387. doi: 10.1186/s12884-021-03862-x (PMC8132374; doi:10.1186/s12884-021-03862-x)
Supplement: Supplementary file 1 — Additional file 1. [file 12884_2021_3862_MOESM1_ESM.docx]

## **Annex: English version questionnaire**

**Socio-demographic and obstetric characteristics of induced women in public hospitals of Keffa, Sheka and Bench-Maji Zone, Southwest Ethiopia, 2018.**

| \| **Part I: Socio- demographic characteristics** \| \| --- \| | | | |
| --- | --- | --- | --- | --- |
| S/No | Questions | Response options | remark |
| 101 | How old are you? | __________Years old |  |
| 102 | Where is your Residence? | 1. Urban  2. Rural |  |
| 103 | What is your religion? | 1. Orthodox  2. Protestant  3. Muslim  4. catholic  5. Others specify------ |  |
| 104 | What is your marital status | 1. Married  2. Not married  3. Widowed  4. Divorced |  |
| 105 | \| What is your educational status? \| \| --- \| | 1.Unable to read and write  2. Able to read and write  3. Primary(1-8)  4. Secondary and above |  |
| 106 | \| What is your current occupation? \| \| --- \| | 1. Gov’t Employee  2. House wife  3. merchant  4. Others specify…… |  |
| 107 | \| To which Ethnic group do you belong? \| \| --- \| | 1. Bench  2. Kafficho  3. Shakacho  4. Amhara  5. Oromo  6. Tigre  7. Others specify…… |  |
| 108 | monthly family income | ___________birr |  |

| \| **Part II: information about Obstetric factors** \| \| --- \| | | | |
| --- | --- | --- | --- | --- |
| 201 | Did you give birth previously before this pregnancy after 7 months of gestation( parity) | \| 1. Yes 2. No \| \| --- \| | If no jump to Q205 |
| 202 | Does your last child alive? | 1. Yes 2. No |  |
| 203 | Did you had faced any obstetric complication in the previous pregnancy | 1. Yes 2. No | If no jump to Q205 |
| 204 | What was that pregnancy related complication in the previous pregnancy | 1. Abortion  2. Stillbirth/IUFD  3. preterm birth  4. Others specify |  |
| 205 | Gestation age of this pregnancy | in weeks |  |
| 206 | Indication(s) for Induction, | 1. Post term  2. PROM ­­­­­­­­­­­­­­  3. Hypertensive disorders  4. Diabetes  5. IUGR  6. APH  7. Others specify |  |
| 207 | Cervical dilatation before starting of induction | in cm. |  |
| 208 | Bishop’s score before starting of induction |  |  |
| 209 | Method of induction (indicate) | 1. Amniotomy  2. Balloon catheter  3. Sweeping membrane  4. Intravenous Pitocin infusion  5. Misoprostol |  |
| 211 | If Intravenous Pitocin infusion used, what is the dose | MIU/ minute |  |
| 212 | If misoprostol only used, route of administration | 1. Vaginal  2. Oral 3. Sublingual  4. Others specify |  |
| 213 | Total amount of misoprostol given | __________________ug |  |
| 214 | Duration of induction of labor | in hours |  |
| 215 | Duration of induction of labor | 1.latent phase labor in hours  2. active phase labor in hours  3. second stage labor in hours |  |
| 216 | Outcome of induction of labor | 1. Success 2. failure |  |
| **Part III: information about maternal prenatal and postpartum factors** | | | |
| 301 | Maternal weight | In kg. |  |
| 302 | Maternal height | In meter. |  |
| 303 | Newborn birth weight | In gram. |  |
| **Part IV: information about outcome of induction of labor** | | | |
| 401 | Uterine hyper stimulation present | 1. Yes 2. No |  |
| 402 | Fetal heart rate non-reassuring following induction of labor | 1. Yes 2. No |  |
| 403 | Change of color of liquor to meconium stained | 1. Yes 2.No |  |
| 404 | Mode of delivery | 1. Normal vaginal delivery  2. Assisted vaginal delivery  3. Caesarean section |  |
| 405 | If delivery by caesarean section, what was the indication: | 1. Failed induction of labour  2. Cephalopelvic disproportion  3. Fetal distress  5. Others indicate |  |
| 406 | Uterine rupture | 1. Yes 2.No |  |
| 407 | Stillbirth | 1. Yes 2.No |  |
| 408 | APGAR score At 1 minute | _______________ |  |
| 409 | APGAR score At 5 minutes | ________________ |  |

THE END

Thank you !!!
